# Feeding, Emotion, and the Brain Stem: The Interesting Case of the Mesencephalic Trigeminal Nucleus

**DOI:** 10.3390/brainsci16010061

**Published:** 2025-12-31

**Authors:** Oliver H. Turnbull

**Affiliations:** 1 North Wales Medical School, Bangor University, Bangor LL57 2DG, Wales, UK; o.turnbull@bangor.ac.uk; 2 School of Psychology, Bangor University, Bangor LL57 2DG, Wales, UK

**Keywords:** brainstem, trigeminal, mesencephalic, emotion, feeding, oral, pleasure, aggression

## Abstract

Background: Our growing understanding of the brain basis of mind has seen an interest in evolutionarily ancient structures, most notably the brainstem. This paper offers an interesting example of this underexplored territory, by considering the mesencephalic component of the trigeminal nucleus. This largely uncelebrated brainstem structure is central to control of the jaw, and for the foundational acts of eating, oral exploration, and biting. Objectives: This paper explores the interesting anatomy of the mesencephalic trigeminal: unique in the nervous system as a centrally located sensory ganglion, which combines sensory and motor function for the jaw. An unexplored aspect of its anatomy is that the mesencephalic component of the nucleus lies directly adjacent to the brain’s core system for the experience of emotion, the peri-acqueductal gray (PAG). Results: The data suggest a role for the jaw, and more broadly the oral cavity, in relation to a range of feeling states, from pleasure to aggression. This is supported by behavioural and classic neuropsychological findings, such as the Klüver-Bucy syndrome. However, the proposal is not well-supported by findings of direct connections between the trigeminal nucleus and the PAG. Conclusions: While these contrasting findings present a conundrum, there may be a role for non-synaptic signalling, of the sort increasingly understood to be important for interoception and homeostasis.

## 1. Introduction

Neuropsychology has, by tradition, been especially interested in various parts of the cerebral cortex [1,2,3,4]. In some respects, this is understandable, given that the cortex, or the cerebral hemispheres more broadly, often appear to be the neural basis for the most impressive aspects of human cognition: language, semantics, decision making, and the like. Indeed, the cortex was, for many years, also believed to be the site of consciousness [5,6].

However, the last few decades have seen an increasing interest in more evolutionarily ancient structures. This has produced a sea-change in our understanding of the brain basis of mind, most notably in relation to the brainstem. Interestingly, this recent change is built on findings that were well-established in neuropsychology but had not been brought together.

Most notably, since the middle of the last century [7], it has been clear that the upper brainstem is responsible for the generation of consciousness, produced by a range of structures usually labelled as the reticular formation, or related terms [8,9,10,11]. This brainstem role in consciousness also seems to be related not to cognition, but to the homeostatic or interoceptive focus of core consciousness [5,6,8,9,10,11].

Conscious experience also seems to map to the role of the brainstem in basic emotions, including from the emotion-related nuclei which produce neurotransmitters such as dopamine and serotonin, in contrast to the deployment of GABA and glutamate across the forebrain in the service of cognition [9,10]. As regards emotional experience, the brainstem is especially important as the site of the periaqueductal gray (PAG), increasingly well-established as the most foundational (though of course not the only) brain region for generating affect [9,10,12]. Notably, recent findings suggest that the PAG has a central role in connections across the upper brainstem, including substantial connectivity to the mesencephalic reticular formation [3], bolstering the argument that emotion and core consciousness are closely related.

These findings suggest that the brainstem is potentially of enormous importance for neuropsychology, and that we should pay greater attention to this critical structure. However, the neuroanatomical training of neuropsychologists has focused on more recently-evolved brain structures, such as the cortex, thalamus, basal ganglia, and hippocampus [13]. In contrast, the brainstem, its nuclei, and their associated cranial nerves, are structures that, in parody: ‘only neurologists and neurosurgeons need to learn about’, and then only in order to understand variants of brain-stem stroke, or surgical approaches to brain-stem tumours.

However, if we are to better understand the origins of the most ancient parts of the mind, we would do well to look more closely at the anatomy of this densely packed and complex brain structure. This paper is one example of a way in which the anatomy of the brainstem may be of relevance for neuropsychology. It suggests that the location and relationships of a previously uncelebrated brain-stem structure may be important for a core concept related to early child development: namely the importance of the jaw, and the oral cavity more generally, in the foundational act of feeding, the pleasurable elements of eating, and perhaps also in acts of oral aggression.

The core argument is that there is an interesting and unusual arrangement in brainstem nuclei that, uniquely amongst body parts, has the potential to link foundational emotional experience and the oral musculature. The argument has four elements:

1. That the anatomy of the trigeminal nucleus is unusual, in that it extends through the entire brain stem, connecting diverse sensory systems.
2. That the function of the trigeminal nucleus is unusual because–despite its primarily sensory role for the entire anterior head, it is responsible for just one highly focused element of motor activity for the face: specifically for oral movements, through the muscles of mastication.
3. That the anatomy of the mesencephalic component of the trigeminal nucleus is unique in the body, because it serves as an internal ganglion, responsible for proprioception for the jaw and oral cavity. This positioning of the nucleus relates to the initial development of the jaw, in the earliest stages of vertebrate evolution.
4. That the mesencephalic component of the trigeminal lies directly adjacent to the brain’s core system for the experience of emotion, suggesting a role for the jaw and oral cavity, uniquely amongst body parts, in relation to feelings, presumably of pleasure and unpleasure.

Below, these four arguments are laid out in more detail.

## 2. The Anatomy of the Trigeminal Nucleus and Sensation

The trigeminal nucleus is unusual amongst brainstem nuclei because of its extensive length. Notably, it is the only brainstem nucleus which extends effectively from the caudal medulla all the way through the pons (where the largest part of the nucleus is found) to the midbrain. Indeed, the trigeminal nucleus extends to the cervical spine and is arguably contiguous with a structure in the spinal cord—the substantia gelatinosa of Rolando. This lies in the dorsal horn of the spinal cord, in the somatic sensory quadrant of the spinal gray matter. The extension suggests a function of connecting, or serving, diverse systems, and is a reminder of the trigeminal’s importance through a long evolutionary history; from the early development of the medulla in relation to homeostasis, to the externally facing systems of the midbrain.

The trigeminal nucleus is of course primarily connected to the trigeminal nerve, the fifth (typically labelled using the Roman numeral V) of the cranial nerves. The nerve is famously divided into three component parts (labelled V_1_ for the ophthalmic, V_2_ for the maxillary, and V_3_ for the mandibular divisions): hence the name, where the pair of left and right nerves are ‘trigeminal’ (=tri-gemi = triplets).

What then is the function of the nucleus? The trigeminal nerve is of the ‘mixed’ type, involved in both motor and sensory activity. However, the vast majority of its function is sensory: responsible for general somato-sensory afferent fibres (i.e., light touch, temperature, vibration, etc.) for almost all the anterior aspects of the head. That is: the external surface of the face (through V_1_, V_2_ and V_3_), the upper surface of the oral cavity (though V_2_), the floor of the oral cavity (through V_3_), and the anterior ^2^/_3_ of the tongue (also through V_3_).

The principal sensory nucleus (V_p_), and the largest part of the trigeminal complex, lies in the mid-pons, and receives discriminative sensation, light touch, and pressure for the face, as well as some proprioceptive information about jaw position.

The trigeminal nucleus therefore provides sensory feedback about the progress of actions relating to movements of much of the face, for activities as diverse as facial expression, speaking, and of course, feeding. All are, of course, activities of evolutionary importance. However, some elements, such as facial expression and vocalisation, are quite recent evolutionary gains, in primates and more widely across mammal species. Feeding, on the other hand, has much more ancient evolutionary origins. In brief, smiling and talking are ‘nice to haves’, but biting and chewing are a life-or-death issue.

## 3. The Trigeminal Nucleus and the Jaw

It is notable that the motor functions for the rest of the anterior head are, with one exception, managed by quite different nuclei, and hence cranial nerves. Most important of these so-called pharyngeal arch, or branchial motor, structures is the facial nucleus, which is the principal (but not only) source of the facial cranial nerve (VII).

It is of interest that there are one set of muscles in the anterior head which are not managed by the facial nerve but instead travel via the trigeminal. These are the so-called muscles of mastication: temporalis, masseter, and the two pterygoid muscles (medial and lateral). All are, in various ways, responsible for closing the jaw, and to some extent moving it backwards, forward, and side-to-side.

What might be so important about the musculature related to feeding that it is placed at the heart of an otherwise sensory nerve complex? As often happens in brain anatomy, there is particular advantage to be gained from having the motor system located close to the relevant sensory inputs. The most famous example of this is at the cortical level, where the primary motor and sensory systems (M_1_ and S_1_) lie adjacent in the pre- and post-central gyrus. However, for almost all of the activities of the anterior face, there is no close mapping in terms of nuclei or cranial nerves between motor and sensory systems. The movements of the extraocular muscles, the upper eye lid, and pupillary dilation, etc., are served by cranial nerves III (ophthalmic), IV (trochlear) and VI (abducent). The muscles of facial expression (apart from the upper eyelid) are served by VII (facial), with few close sensory connections. Movements of the tongue and associated pharynx and larynx are run by IX (glossopharyngeal), X (vagus), XI (accessory) and XII (hypoglossal), again without close motor-sensory connections.

In sum, the trigeminal is unusual in controlling both motor and sensory activity for the process of mastication and related oral movements. It manages the motor element of this process in the central part of the trigeminal nucleus—the main body of V, located in the pons. As discussed above, the trigeminal also has upward and downward extensions. It is the upward extension which may be of particular interest to neuropsychology.

## 4. The Mesencephalic Component

The upwards extension of the trigeminal, the so-called mesencephalic component of the nucleus, is highly unusual within brain stem anatomy. It is responsible, importantly, for sensation relating to opening and closing the jaw. This is achieved primarily through the stretch receptors for the muscles of mastication, as well as for pressure and kinaesthetic sensation for the teeth, periodontium, and the hard palate [14]. Essentially, this component of the nucleus provides proprioceptive feedback from the structures which surround the oral cavity (including the anterior ^2^/_3_ of the tongue itself). These have long been understood to control the position and strength of the bite, and the management of activities such as chewing and sucking [15].

What makes the mesencephalic component of the trigeminal unique? This is because the mesencephalic extension is technically not a classic nucleus, but a sensory ganglion, of the sort that is normally found outside, rather than inside, the brainstem. Neuroanatomists raise this issue repeatedly. It is the single exception which breaks an otherwise standard pattern of neural organisation: “Sensory neurons have their cell bodies outside the central nervous system, except the mesencephalic nucleus of the trigeminal nerve” [16]. Authors describe the nucleus as “unique” [17]; “a very unusual nucleus” [18]; “one of the most distinct collections of cells in the central nervous system” [19], underpinning the question: “Why is the mesencephalic nucleus of the trigeminal nerve situated inside the brain?” [20]. This exceptional anatomical arrangement is true of almost all vertebrate species (see below for the exceptions). It suggests that there was some special advantage in the (evolutionarily) early incorporation of the system for oral proprioception into the brainstem, as opposed to representation of other parts of the head (see [20] for speculation about this issue).

Why is this true of almost, but not all, vertebrates? The exception is the absence of this internal ganglion in the lamprey and hagfish—the only vertebrates without a jaw [15,21]. It is only with the presence of a jaw, and the associated muscles required for mastication, that there is a need for a unique sensory source for the oral cavity [22,23,24].

In sum, we have a brainstem nucleus which is uniquely located because of its importance in the control of the vital process of feeding, introduced in early vertebrates to support the innovative development of the jaw. The sensory aspect of this masticatory process is managed by the mesencephalic (or midbrain) component of the trigeminal, while the motor elements are managed in the motor nucleus, located in the pons.

## 5. The Mesencephalic Component and Emotion

What then can we say of the importance of the midbrain in relation to other activities, particularly of those linked to conscious mental life? The caudal parts of the brainstem, the medulla and lower pons, are well-understood to manage a range of homeostatic or interoceptive control systems: the control of respiration, baro- and chemo-reception, temperature regulation, and the like. Notably, these appear to operate outside of conscious awareness [5,6,9,10,25,26,27].

However, the upper (rostral) pons and midbrain are the sites of the various nuclei of the reticular formation, which we know are at the heart of conscious awareness [5,6,7,9,10]. But consciousness of what? We know that the structures in a particular part of the midbrain are at the heart of the brain’s basic emotion systems: the periaqueductal gray (PAG), which was identified as core to emotional experience [5,8,10,12]. It lies (as the name suggests) surrounding the cerebral aqueduct, in the posterior midbrain [28]. It is especially powerful as an evoker of forms of defensive behaviour [29,30], and is the brain area from which emotion “can be most readily evoked in humans and animals with the lowest levels of brain stimulation” [31]. Indeed, several authors have suggested that this region (and related structures) might be the core of the ‘self’ [6,12,29].

Recall that the trigeminal nucleus is primarily sensory in nature and has a mesencephalic extension. It is of particular interest that this mesencephalic component is sensory, but only for mastication, and hence control of sucking, biting, and chewing in the oral cavity. Critically, it appears that this mesencephalic extension lies adjacent to (i.e., it is a direct anatomical boundary of) the peri-aqueductal grey [15,32].

What advantage would there be in co-locating a sensory system for one part of the head, with the brain’s central emotion generation system? Presumably by generating associations between movements of the oral cavity and emotional experience, so that actions of the jaw and oral cavity can be experienced as pleasurable or aversive. We investigate this possibility, firstly through a review of neuropsychological and other behavioural data, and secondly by a survey of the anatomical projections to the PAG.

## 6. Neuropsychological and Other Behavioural Sources

As mentioned above, there is a long-standing awareness of the importance of pleasurable sensations related to the mouth and feeding in early development [24,33], with an explicit link to the evolutionary advantages of this phenomenon [33]. This includes the complex issue of sensation from the mouth, including the multi-sensory nature of the oral experience in eating [34]. Stand-out examples include complex (viscosity-based) somatosensation in response to the mouth-feel of fatty textures, which are notably distinct from the pleasure experienced from sucrose (involving, for example, separate parts of the insula) [34]. These oral experiences are also powerfully affective and readily produce pleasurable and aversive conditioning [34].

We might also address the question of pleasure related to the mouth in adults. Quite apart from a role in eating, adults engage in social actions with the mouth, most notably kissing (and other erotic activity). Oral intimacy is a human universal, also reported across historical time periods [35,36,37], and shows continuity of behaviour with our large ape common ancestors [36].

The mouth is also involved in powerfully negative feelings, again mediated by the PAG. The most obvious examples are feelings of rage [12,31]. During angry and defensive states, animals (including very young or disinhibited humans) have a powerful desire to bite their opponents [12,38]. As the infant develops, and acquires emotion regulation [38,39], this biting continues, but is contained, to avoid damage to others. This managed oral aggression is seen, most famously, in rough-and-tumble play [12]. Indeed, we can see the central role of the jaw in expressions of anger even when the opponent is not present. When adult humans merely pretend to adopt an enraged posture, they produce clenching of both the fists and the jaw—reminding us that the jaw is a core and prominent feature of the RAGE action pattern [12,40].

The most obvious neuropsychological manifestations of these phenomena appear in Klüver-Bucy syndrome [41,42]. Classic Klüver-Bucy follows from bilateral lesions to the anterior temporal lobes and related structures, including the amygdala. The result is a remarkable combination of hyperorality, hypersexuality, aggression and visual agnosia (mouthing objects that are not visually recognised).

Marlowe et al. [41] describe a classic case (after encephalitis of presumed herpetic origin): “He engaged in oral exploration of all objects within his grasp, appearing unable to gain information via tactile or visual means alone. All objects that he could lift were placed in his mouth and sucked or chewed. He was commonly observed to place his fingers in his mouth and suck them… he ingested virtually everything within reach, including the plastic wrapper from bread, cleaning pastes, ink, dog food, and feces” [41].

The most likely explanation for these phenomena seems to be damage of a range of forebrain regulatory functions. The lesions ‘release’ (to use a Jacksonian term) or disrupt the ‘balance-against’ [43] mechanisms discussed above, re-prioritising the mouth as a source of exploration, pleasure, and aggression. An analogue is the developmental importance of the mouth in younger children, which can continue in those with profound learning disabilities. In these developmental cases we also have a great reliance on oral exploration, again indiscriminate in nature, and associated with poorly regulated anger [38,44,45,46].

In sum, the oral cavity is deployed in a range of basic emotions. The extent to which these actions are pleasurable or unpleasurable, and the mechanism of their regulation, is beyond the scope of this paper. Regardless, it seems clear that the oral cavity plays an important role in emotion, even more widely than the pleasures associated with food.

However, this evidence, even from Klüver-Bucy, is indirect. These neuropsychological and behavioural data suggest only that the ‘release’ of powerful innate brainstem mechanisms from cortical or limbic control produces powerfully motivated oral behaviour. That alone does not suggest that it is a direct relationship between the mesencephalic trigeminal to the PAG that drives the process, though an upper brainstem mechanism seems a plausible candidate. To investigate that issue, we review the inputs to the PAG.

## 7. Inputs to the PAG

Is this anatomical neighbourliness also supported by findings of connectivity between the mesencephalic trigeminal and the PAG? Like so many brain regions, the PAG receives projections from a wide range of sources.

A primary descending route is from a number of frontal regions, especially the medial and orbital (but not the lateral) surfaces [28]. Notably, projections from the posterior cortex (and thus sensory systems) are absent at this level, as are projections from the hippocampus and basil ganglia [28]. These medial frontal connections presumably reflect the most evolutionarily recent, and later-developing, inputs to the PAG, especially associated with the regulation of emotion [47].

A second source of input to PAG is the extended amygdala: especially from the central nucleus and the ventrolateral part of the basal nucleus. Interestingly, the ventrolateral part of the basal nucleus itself projects back to medial prefrontal cortex. These amygdaloid projections to PAG are less extensive than those from the frontal cortex [28], and this pathway is typically interpreted as underpinning the role of the PAG in negative emotions such as anger and fear [48].

An especially powerful source of inputs to the PAG is from the hypothalamus, especially the medial preoptic and the anterior hypothalamic regions, the periventricular dorsal, ventromedial, and periarcuate hypothalamus. There is little input from the supraoptic, suprachiasmatic, and paraventricular regions [28]. Again, these regions carry autonomic information to the PAG and may represent another emotion-related source.

At the level of the brainstem, there is a rich pattern of inputs to PAG, including from both colliculi. There are also extensive connections with nuclei associated with consciousness, such as the mesencephalic reticular formation and the parabrachial nucleus [9,10,28]. As well as projections to the PAG, these sites also receive projections from the PAG, presumably as part of the core systems which underpin consciousness and arousal [9,10,28]. There are also interesting connections to the nucleus of the solitary tract, which presumably gathers visceral (chemo and baro-sensory) information for the PAG [29].

Most importantly for our purposes, there are also some inputs to the PAG from the trigeminal nucleus. However, these are actually rather modest as regards the mesencephalic trigeminal, though this has not been a core focus of investigation. However, there is evidence of PAG projection from proprioceptors signalling chewing activity (which initiate histamine-receptive neurons in the mesencephalic trigeminal) and then activate hypothalamic cells [14].

The most substantial projections come not from the mesencephalic component to PAG, but rather from the *spinal* component of the trigeminal complex. Indeed, the PAG is “almost the sole target of the projection from the laminar spinal trigeminal nucleus” [49]. This spinal component is an ancient part of the trigeminal system, and the spinal-PAG projection is (like the PAG itself) highly conserved across species, much more widely even than mammals [49]. The spinal trigeminal nucleus carries the most primitive forms of sensation, especially focusing on noxious stimuli: nociceptive and thermoreceptive information [28]. Importantly, the spinal nucleus projects especially to the lateral region of the PAG, which controls defensive behaviour, especially in relation to the face and forelimbs [49], presumably so that threats to bodily integrity are directly transformed into powerfully averse feelings, a finding recently supported by imaging data [50]. To give an impression of the scale of this spinal-trigeminal to PAG projection, it has some three times as many neurons as the much better-known spinothalamic projection [51]. Apparently, it is quite important to know *where* I am in pain, but it is (three times) more important to know that *I am in pain*!

## 8. A Solution to the Conundrum?

What then of our hypothesis? We are faced with two contradictory findings. The mesencephalic nucleus of the trigeminal is clearly an anatomically remarkable structure, important for the control of oral movements, and lies directly adjacent to a critical upper brainstem structure for the experience of emotion. When released from cortical control, this brainstem system appears to produce (in Klüver-Bucy syndrome) a powerful desire to explore objects with the mouth, in a manner reminiscent of (some features of) early child development.

On this basis, we would expect substantial projections from the mesencephalic trigeminal to the PAG. However, these are not especially prominent, and the trigeminal connections to the PAG are instead primarily from the spinal component, notably in relation to bodily threatening noxious stimuli.

How might we reconcile this contradiction? One possibility is insufficient evidence. All authorities agree that the PAG, its function, and its connections, represent an important but understudied topic [10,11,12,14,28,31,47]. Thus, there may be an as-yet undiscovered solution of the mesencephalic trigeminal-PAG connection.

An interesting possibility might come from our growing understanding of the way in which interoceptive systems in general might rely on ‘analogue’, unmyelinated, or non-synaptic signalling [11]. It may well be that neural *projections* to the PAG are not the correct unit of currency, and that other sources of neurochemical transmission are important [14].

For non-synaptic interactions, anatomical proximity is vital, achieved by integrating physiological cues from nearby tissue [11]. Of course, the mesencephalic trigeminal is literally one of the boundaries of the PAG, so the two structures share a microenvironment. This offers an opportunity for ‘volume transmission’, or ambient extracellular diffusion, as a signal source. This mechanism is increasingly well-described as being deployed for various classes of interoception [11], and the PAG would be an interesting candidate.

Other mechanisms of non-synaptic interaction have been described but seem less likely for our case. Ephaptic coupling (electric field effects between neurons) is most clearly seen in (lightly myelinated) fibre bundles, such as the vagus nerve, for which the mesencephalic trigeminal to PAG case is not a candidate example. A final non-synaptic class are fenestrations in the blood–brain barrier, of which the prototype are the circumventricular organs (such as the area postrema). It is of some interest that the peri-acqueductal gray is—literally in its name—‘circumventricular’. However, the PAG does have a blood–brain barrier, and in any case this is not relevant to the mesencephalic trigeminal to PAG interaction.

Thus, volume transmission, by ambient extracellular diffusion in a microenvironment, would be the most likely non-synaptic signal source. This would make the anatomical proximity of the mesencephalic trigeminal and PAG a design feature, rather than an anatomical accident. I am not aware of any research on this topic, and clearly any class of non-synaptic interaction here would form an interesting line of future research.

A second alternative is that we have the correct phenomena, but the wrong brain structures. On this argument, the link between emotion and the mouth remains powerful and important, in settings as diverse as feeding, kissing, and biting, made manifest in the hyperorality of the Klüver-Bucy syndrome. However, the proximity of the mesencephalic trigeminal to the PAG may not be the anatomical origin of these important phenomena. Potential target structures would presumably need to involve a link between emotion systems and oral sensation/musculature, and an interesting short list could be developed. Candidates might include the amygdala, hypothalamus, septal nuclei, or even the nucleus of the solitary tract, by analogy with other instances of powerful emotions generated by subcortical brain regions [52].

## 9. Conclusions

This example issue is a reminder for neuropsychology of the importance of the brainstem in relation to the mind. Here we have structures which form in utero [53,54,55] and progressively link medullary nuclei (that underpin homeostasis) with midbrain and pontine nuclei (that mediate consciousness and other aspects of the mind) [9,10,52]. This developmental process will likely also include neurochemical interactions directly from the viscera, including from the enteric nervous system [11]. The under-investigated question is *how* these communications occur, at which stage of intrauterine development, and their long-term effects on developmental variation and pathology. These are questions with important scientific and clinical consequences (notably in psychiatry and child development) and also bear on significant ethical issues. Their anatomy may even show us how to construct artificial consciousness, should one want to [6]. Plainly, these are important issues for modern science, and we would do well to dust off our brainstem neuroanatomy in trying to address them.

## Data Availability

The original contributions presented in this study are included in the article. Further inquiries can be directed to the corresponding author.
